# Supplementary material for: DNA Hypomethylation in the TNF-Alpha Gene Predicts Rheumatoid Arthritis Classification in Patients with Early Inflammatory Symptoms
Source: Cells. 2023 Sep 28;12(19):2376. doi: 10.3390/cells12192376 (PMC10571942; doi:10.3390/cells12192376)
Supplement: Supplementary file 1 [file cells-12-02376-s001.zip › cells-2590053-supplementary.pdf]

Supplementary file

Assay principle

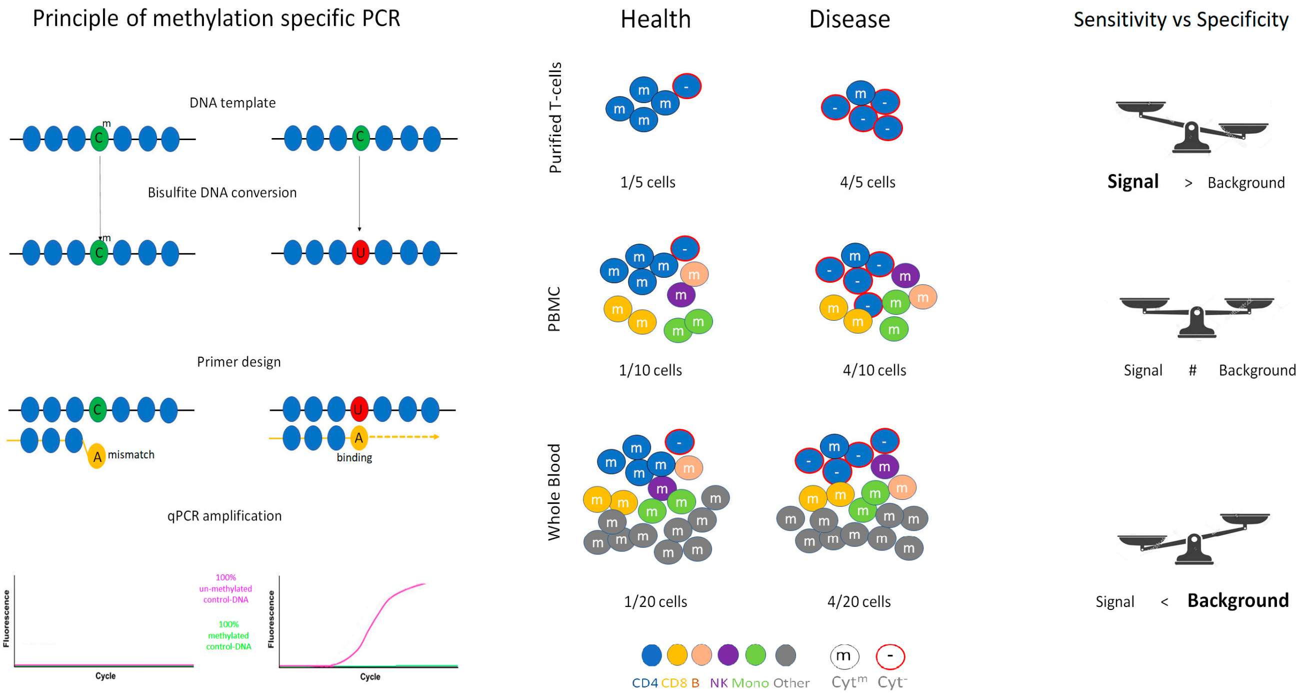

To differentiate between methylated cytosine (C^m) and unmethylated cytosine (C) by PCR, bisulfite conversion of the DNA is necessary to provide a template where these can be differentiated by changing all C into an Uracil but keeping the C^m untouched **(top left panel)**. Designing the qPCR assay then relies on the same principles as other qPCR assays based on using primers to ensure the specificity of the PCR product and a probe to quantify it **(bottom left panel).** Methylated DNA (100% control) should not generate a product while 100% control de-methylated DNA will yield a PCR amplification.
Methylation at a CpG site is a binary event, so the ratio of cell with methylation to cells without methylation will be measure as levels of methylation (%) in the total population of cells analysed. In purified T-cells, patients will ideally exhibit cells with unmethylated-DNA, while healthy controls will have Methylated-DNA (**top middle panel**). The signal will therefore be high and the background should be low (**top right panel**). In PBMC, T-cells represent only 50% of the PBMC, so the signal will be diluted (+1 Cycle difference for 50% T-cells) and the background will increase (-1 cycle for 50% non-T-cells). Balancing both will determine if the assay can be developed and a signal can be detected between health and disease. In whole blood, where more cell types dilute the signal even further (+2-3 cycle compared to T-cell), it will only be possible to detect the signal if it is very high compared to the background so with a very large difference between health and disease.
qPCR being very sensitive all type of assay can work but the frequency of the cell of interest, the amplitude of the signal and the local sequence will be determinant in the success of the design. For example Treg can be detected because the CpG in the FoxP3 gene used to design the assay is ONLY demethylated in Treg, even if they are a small fraction of CD4+T-cells in PBMC and in WB, none of these other cells being able to generate a PCR product.

SUP Table S1 : datasets used for the selection of CpG candidates

Dataset ID	Cells type	samples	technology
GSE121192	• Naïve CD4 T-cells<br>• Memory CD4+Tcells<br>• Monocytes	6 HC,<br>10 early RA	450K bead array
GSE71841	CD4+T-cell	12 HC	450K bead array
GSE35069	CD4+T-cells,<br>CD8+T-cells,<br>NK cells,<br>B-cells,<br>Monocytes,<br>Neutrophil,<br>Eosinophil,<br>Granolocytes	6 HC	450K bead array
GSE71841	CD4+T-cell	12 HC,<br>12 RA	450K bead array
GSE 111942	PBMC	18 HC,<br>25 RA	450K bead array
GSE 87095	B-cells	73 HC,<br>49 RA	450K bead array
GSE 117929	PBMC	19 HC,	450K bead array
GSE 82218	PBMC	25 HC,	450K bead array
GSE 88824	WB	14 HC,	450K bead array
Personal communication	Naïve CD4+T-cells<br>Monocytes,<br>B-cells	31 HC,<br>63 RA	450K bead array

SUP Table S2 : Top ranking of CpG candidate

Selection strategy 1

**Selection of candidate CpG**		Naïve CD4+T-cells$				Other lymphocytes Median β-value *				Other Whole blood cell types Median β-value (%)*				
CpG	Gene	Medianβ-value HC$	Medianβ-value RA$	Δβ-value $	p-value$	CD4+T-in HC	CD8+T-in HC	B-cells in HC	NK-cells in HC	Mono in HC$	Mono in RA$	Neutrophil in HC	Eosinophil in HC	Granolocytes in HC
**cg17741993**	**TNF**	0.53	0.30	-0.22	0.0005	0.27	0.34	0.43	0.17	0.87	0.90	0.86	0.90	0.84
**cg16379091**	**IFITM1**	0.57	0.37	-0.20	0.0002	0.60	0.46	0.88	0.44	0.90	0.89	0.89	0.90	0.87
cg13681468	GPRIN3	0.49	0.17	-0.32	0.0005	0.44	0.35	0.84	0.33	0.90	0.92	0.90	0.91	0.88
cg03718883	INS-IGF2	0.59	0.35	-0.24	0.0004	0.59	0.59	0.79	0.70	0.82	0.83	0.84	0.85	0.84
cg05246522	KSR1	0.39	0.17	-0.22	0.0002	0.38	0.32	0.45	0.27	0.84	0.82	0.72	0.50	0.80
cg20105257	HLA-E	0.45	0.25	-0.20	0.0002	0.42	0.30	0.89	0.64	0.98	0.98	0.96	0.97	0.94
cg00759807	LOC100287036	0.57	0.37	-0.19	0.0003	0.37	0.42	0.53	0.39	0.94	0.93	0.93	0.94	0.89
cg27183791	ANKRD11	0.50	0.32	-0.18	0.0000	0.25	0.14	0.69	0.07	0.82	0.77	0.76	0.77	0.79
cg05784862	KSR1	0.36	0.18	-0.18	0.0006	0.37	0.33	0.46	0.30	0.80	0.77	0.71	0.57	0.74
cg02231590	ITM2C	0.54	0.37	-0.18	0.0001	0.51	0.42	0.06	0.46	0.84	0.82	0.81	0.83	0.82
cg00576086	TERT	0.55	0.38	-0.17	0.0001	0.66	0.35	0.81	0.57	0.84	0.83	0.86	0.86	0.83
cg25256924	PTPRCAP	0.39	0.24	-0.15	0.0009	0.31	0.33	0.55	0.44	0.88	0.88	0.77	0.74	0.80
cg17851795	PBX2	0.59	0.45	-0.15	0.0005	0.66	0.59	0.76	0.71	0.83	0.82	0.80	0.80	0.79
cg12669088	KRAS	0.39	0.24	-0.14	0.0008	0.43	0.39	0.61	0.40	0.86	0.85	0.74	0.39	0.81
cg05299836	BCKDK	0.46	0.32	-0.14	0.0005	0.54	0.47	0.52	0.49	0.83	0.82	0.81	0.80	0.78
cg01260502	GIMAP7	0.32	0.18	-0.14	0.0007	0.55	0.44	0.84	0.69	0.91	0.90	0.91	0.91	0.90
cg03050965	S1PR1	0.57	0.44	-0.13	0.0002	0.69	0.52	0.78	0.49	0.84	0.83	0.80	0.82	0.79
cg23660197	MICB	0.49	0.37	-0.12	0.0003	0.53	0.39	0.75	0.46	0.81	0.78	0.75	0.73	0.77
cg04618171	HPCAL1	0.35	0.23	-0.12	0.0001	0.66	0.71	0.93	0.87	0.92	0.92	0.92	0.92	0.92
cg20703928	NCK2	0.24	0.12	-0.12	0.0006	0.44	0.36	0.83	0.67	0.93	0.93	0.89	0.92	0.89
cg06813419	TRAF5	0.54	0.43	-0.10	0.0007	0.64	0.55	0.61	0.76	0.93	0.93	0.93	0.94	0.92
cg23149454	PDE2A	0.56	0.47	-0.10	0.0002	0.57	0.44	0.76	0.55	0.88	0.88	0.86	0.89	0.84
cg14885762	SEPT9	0.25	0.16	-0.09	0.0006	0.46	0.41	0.12	0.64	0.83	0.82	0.84	0.81	0.83
cg15350899	BCL9L	0.28	0.21	-0.07	0.0006	0.50	0.36	0.81	0.54	0.81	0.79	0.79	0.81	0.77
cg14665366	GPRIN3	0.14	0.08	-0.07	0.0007	0.28	0.18	0.86	0.29	0.94	0.92	0.90	0.90	0.88
cg01105418	ZBTB18	0.40	0.59	0.19	0.0002	0.80	0.73	0.58	0.87	0.83	0.81	0.82	0.71	0.83

Selection strategy 2

**Selection of candidate CpG**		Naïve CD4+T-cells[$]				Other lymphocytes Median β-value *				Other Whole blood cell types Median β-value (%)*				
CpG	Gene	Medianβ-value HC[$]	Medianβ-value RA[$]	Δβ-value [$]	p-value[$]	CD4+T- in HC	CD8+T- in HC	B-cells in HC	NK-cells in HC	Mono in HC[$]	Mono in RA[$]	Neutrophil in HC	Eosinophil in HC	Granolocytes in HC
**cg15058210**	**HDAC4**	0.53	0.35	-0.19	0.0001	0.43	0.32	0.64	0.26	0.10	0.09	0.06	0.05	0.06
**cg15978561**		0.25	0.12	-0.14	0.0005	0.24	0.20	0.59	0.19	0.04	0.04	0.05	0.05	NA
**cg05903736**		0.33	0.21	-0.12	0.0004	0.33	0.29	0.59	0.27	0.12	0.11	0.07	0.06	0.06
**cg12054453**	**MIR21**	0.22	0.11	-0.11	0.0019	0.23	0.19	0.47	0.10	0.20	0.17	0.38	0.32	0.39
**cg24174557**		0.32	0.21	-0.11	0.0004	0.33	0.32	0.17	0.28	0.30	0.27	0.32	0.39	0.29
**cg16936953**		0.40	0.20	-0.20	0.0013	0.33	0.36	0.55	0.25	0.37	0.36	0.61	0.62	0.64
**cg02835823**	**IRF8**	0.28	0.42	0.14	0.0007	0.60	0.45	0.07	0.41	0.63	0.65	0.80	0.84	0.79
cg16853860	PSMB9	0.72	0.51	-0.21	0.0071	0.58	0.38	0.55	0.37	0.74	0.68	0.70	0.63	0.71
cg26427498	NAMPT	0.38	0.20	-0.19	0.0035	0.32	0.24	0.67	0.23	0.05	0.04	0.05	0.04	0.04
cg08752433	PPTC7	0.74	0.56	-0.18	0.0021	0.58	0.49	0.68	0.47	0.62	0.66	0.41	0.27	0.39
cg01106881	DNPEP	0.49	0.32	-0.17	0.0011	0.34	0.23	0.24	0.11	0.06	0.05	0.09	0.10	0.06
cg22077313	S100P	0.61	0.44	-0.17	0.0001	0.47	0.29	0.72	0.19	0.37	0.35	0.20	0.21	0.18
cg13064571	C8orf44	0.54	0.39	-0.15	0.0012	0.48	0.50	0.70	0.23	0.18	0.20	0.10	0.06	0.07
cg00004667	ZBTB17	0.26	0.11	-0.15	0.0002	0.25	0.18	0.03	0.14	0.05	0.05	0.06	0.07	0.04
ch.2.207814544R	KLF7	0.31	0.16	-0.14	0.0001	0.25	0.25	0.02	0.04	0.04	0.04	0.04	0.03	0.04
ch.2.105901354F	NCK2	0.34	0.21	-0.13	0.0013	0.30	0.30	0.02	0.04	0.02	0.02	0.04	0.03	0.03
cg20388732	STAT5A	0.27	0.14	-0.13	0.0075	0.31	0.11	0.26	0.09	0.05	0.05	0.06	0.05	0.06
cg03206537	NEURL2	0.43	0.31	-0.12	0.0005	0.39	0.30	0.40	0.24	0.28	0.27	0.16	0.08	0.17
ch.13.39564907R	LINC00332	0.26	0.16	-0.10	0.0003	0.25	0.25	0.04	0.05	0.04	0.04	0.06	0.05	0.05
cg17002328	CCDC88C	0.71	0.82	0.10	0.0091	0.85	0.76	0.20	0.71	0.85	0.84	0.83	0.79	0.85
cg23458168	ZNF536	0.46	0.58	0.12	0.0072	0.69	0.59	0.56	0.49	0.43	0.52	0.45	0.44	0.48
cg20793665	PTMA	0.34	0.47	0.13	0.0057	0.56	0.50	0.33	0.80	0.88	0.83	0.84	0.87	0.86

[$] our dataset published (39) CpG in bold are those taken forward for qMSP assay development.

SUP figure S1 : Structure of the DNA region surrounding the candidate CpG in the TNF gene. a) from our dataset comparing naïve and memory CD4+T-cell and monocyte in HC and RA, and b) in various blood cell types in health.

a) Methylation levels from our dataset

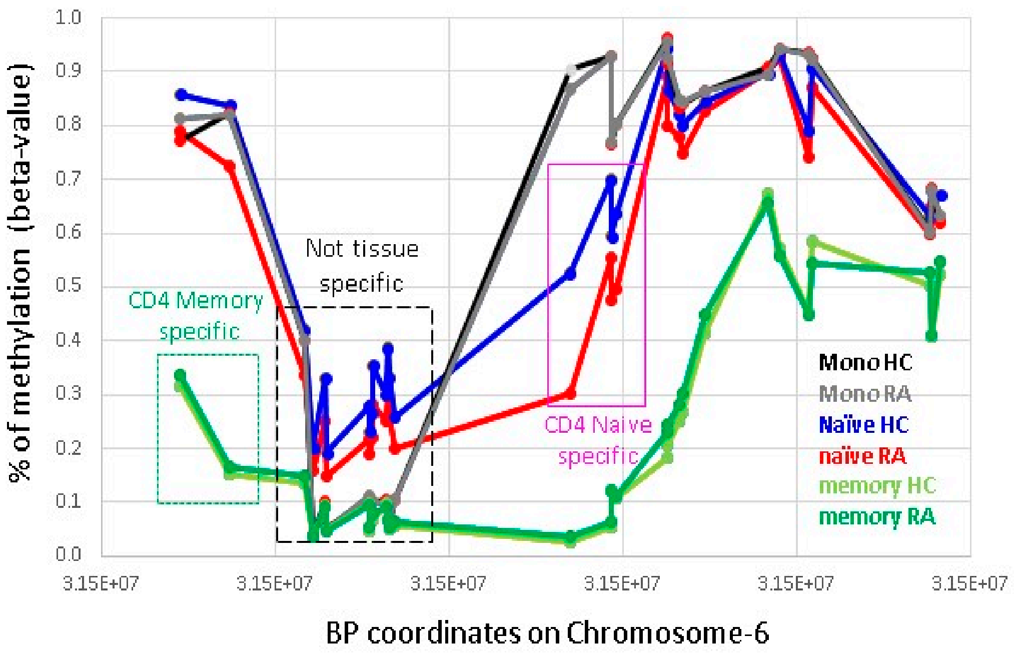

b) Average methylation levels in blood cell types from several datasets (the dotted line represents naïve CD4+T-cells, plain line is memory cells)

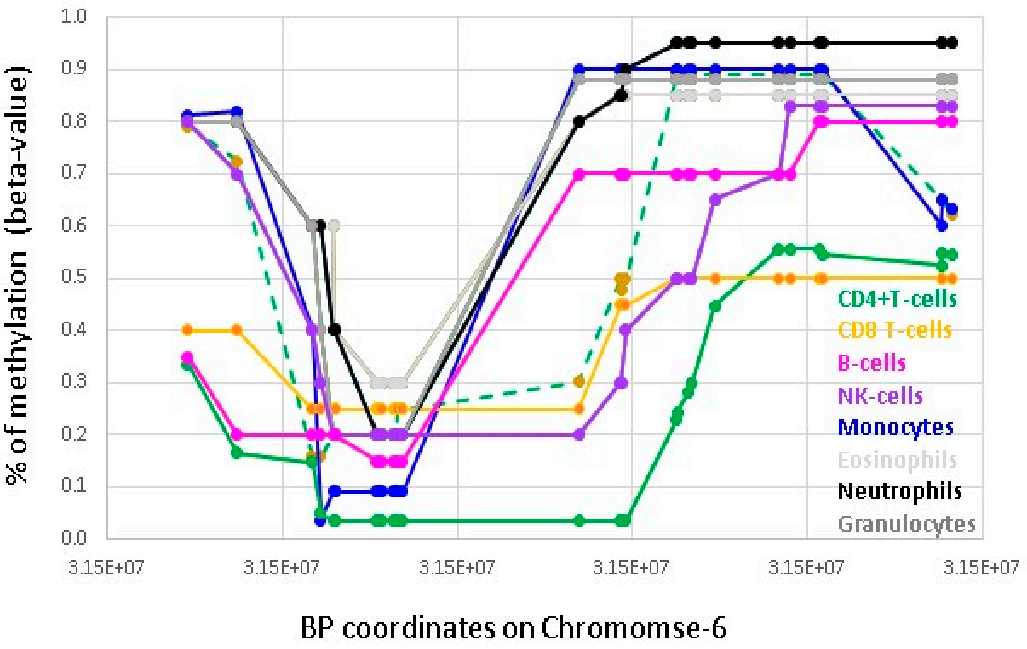

The candidate CpG from the selection process, namely cg11484872 is located on chromosome 6 (GRCh37/hg19) at the coordinates chr6: 31543169. It was identified in the 450K Methylation Array dataset and is part of the promoter region of the TNF gene, approximately 200 base pairs upstream of the transcription start site. The primers and probe designed for the TNFα qMSP assay target the region where this cg11484872 is located and the PCR product include another 4 CpG sites (boxed on the top graph) similarly differentially methylated. In the bottom graph, publicly available data in health for other cell types in the blood. The DNA in the region of interest if methylated in most cells for B-cells, monocytes, eosinophils, neutrophils and granulocytes hence unlikely to interfere with the assay. T-cells (naïve/memory) CD4+T-cells, CD8+ T-cells and NK cells show a higher frequency of cell with demethylated-DNA and this is where the size effect of the differential methylation ($\Delta\beta$) between comparator groups (Health and RA) will determine whether the essay can be designed or not.

Detailed of DNA preparation

Genomic DNA was extracted using a silica-membrane-based DNA purification kit (QIAamp DNA Blood Mini Kit), according to the manufacturer's protocol. Briefly, cell pellets were re-suspended in 200 uL PBS and 20 uL of Proteinase K were added, followed by 200 uL of lysis buffer (Buffer AL) and incubated at 56ºC for 10 min. DNA was precipitated by adding 200 uL of pure ethanol. The sample was transferred to a QIAamp Mini spin column and centrifuged to separate the supernatant at 15000g for 1 min (used for all centrifugations). The DNA, adsorbed on the QIAamp silica membrane in the column was washed twice (wash Buffer AW1 and Buffer AW2), to remove any residual contaminants. Purified DNA was then eluted (Buffer AE) for 10 min before and centrifuged out of the QIAamp Mini spin column. DNA quality and concentration were asserted using a spectrophotometer (ND1000). An absorbance ratio (OD280/OD260) at 1.8 – 2 was accepted as pure DNA.

Detailed Bisulfite conversion of genomic DNA

To differentiate between methylated and un-methylated cytosine by PCR, bisulfite conversion of the DNA is necessary to differentiate unmethylated-cytosine turned into Uracil while keeping methylated-cytosine untouched. Bisulfite conversion was performed using the EZ DNA Methylation-Gold Kit (Zymo Research) following the manufacturer's protocol. Briefly, 500 ng of genomic DNA (adjusted to 20 uL) and 130 uL of the CT conversion reagent were mixed in PCR tubes and placed into a Thermal Cycler (Techne™ TC-512 Gradient Thermal Cycler, Bibby Scientific) programmed at 98 ºC for 10 min and then at 64ºC for 2.5 h. Samples were then transferred to the Zymo-Spin™ IC Column containing 600 uL of binding buffer and centrifuge (10000 g for 30 sec) before desulphonation and clean-up by adding 200 uL of desulphonation buffer followed by several washing and centrifugation steps. Pure Bisulfite-converted DNA was eluted from the column matrix with 20 uL of Elution Buffer and used for methylation analysis. The quantity of DNA after bisulfite converted was measured again by nanodrop.

Detailed Primer design for qMSP

Designing the qPCR assay then relies on the same principles as other fluorescent-probe based assay, using primers to ensure specificity of the PCR product and a probe to quantify it. TaqMan® MGB Probe and primer set were designed using Primer Express Software v2.0. Two types of reactions are needed for a qMSP quantitative assay, one for the methylation-dependent CpG of interest and one for a methylation-independent CpG for a control gene used for normalization.

For the gene of interest, primers were designed to amplify a region next to the candidate CpG site to be tested, according to qPCR primer design guidelines (46-49). Both the sense or antisense DNA strands could be used for designing primers/probes. First, the genomic DNA

sequence of candidate regions was obtained from UCSC or NCBI database (using Human Genome Assembly GRCh37.p13). The sequence was then bisulfite converted *in silico,* using the sequence manipulating function in the online platform, MethPrimer 2.0.
For the candidate CpG, the primer sequences were manually edited to obtain the best Tm (59-60 ºC), positioning the candidate CpG site at 3' end of a primer (either F or R), for a length of the PCR product of ~100 pb, avoiding self-dimers/hairpins or primer-dimers. A probe was also designed to encompass CpG sites where possible. The PCR product was checked *in silico* to ensure the specificity to the gene of interest (absence of homology with other sequences after bisulfite-conversion of genomic DNA), using the blasting function of the Bisearch primer-design and search tool.
For the internal control, the qMSP primers should amplify bisulfite modified DNA independently of its methylation status. Primers/probes were therefore designed to avoid CpG sites.
All primers were synthesised by Thermo Fisher Scientific, reconstituted to a concentration of 100 uM in sterile water. TagMan custom probe-MGBNFQ were synthesised by Applied Biosystems reconstituted to a concentration of 2.5 uM in sterile water. Primers and probe were aliquoted stored at -20ºC. The details of each design are described in the results sections.

Detailed qMSP assay optimisation

The development of qMSP assays starts with reactions optimisation which was done using both 100% methylated and 100% unmethylated control-DNA. qPCR conditions were optimised using a standard PCR mixture as recommend by manufacturer's instructions for TaqMan™ Universal Master Mix-II, varying the concentration of F and R primers. The reaction were performed in the Applied Biosystems™ QuantStudio™ 5 Real-Time PCR System. In order to ensure the specificity (methylated/unmethylated DNA) and efficiency (equivalent yield for each PCR product), each assay was optimised varying primer concentrations from 50-900 nM, Primers being the main factor affecting the efficiency of the qPCR reaction. For this, reactions were set up varying primer concentrations and directly compared using Cts between assays. A dilution series of the template DNA (0.2 to 50 ng) was then used to compare efficiencies of the different assays. The Optimized assay was then used to quantify methylation of the target locus in genomic DNA from patient samples.

Detailed assay discussion

qMSP assay permits the use of blood (or other mixed tissue) as biological template, but this imposed a number of conditions on the suitability of the candidate CpG. The ideal situation would require the methylation status of the candidate CpG in the target cells (here demethylated in CD4+T-cells) to be different from the status in all other cell types (ie fully methylated in all non-CD4+T-cells). This restricts considerably the number of candidates

which also need to be differentially methylated in samples between the outcome of interest and its alternative. The dataset to search for candidate CpG sites was generated in naïve CD4+T-cells identified > 3000? differentially methylated CpGs (39). The *TNF* candidate gene appeared highly relevant to RA and we previously confirmed lower methylation in early RA using sequencing (39). The CpG chosen was in a wider region that is demethylated in T-cells (naïve and memory), while not in monocytes and with mixed pattern in other cell types. The dilution of signal comparing purified CD4+T-cells to PBMC and WB suggests that the higher % of methylation observed results from loss of signal due to methylation in non-CD4+T-cells, while the Δβ-value is still sufficiently sensitive in PBMC over purified cells, while no longer in WB. *IRF8* and *IFIMT1* were selected due to their potential role of IFN-related signalling in pre-RA at the gene expression levels (53-54)and *MIR21* for its differential expression in pre-RA (55) and the abundant literature associating it with RA 56-60), however, designing assays was not successful for the specific CpG chosen for *MIR21* and unsatisfactory for *IRF8*. For the *HDAC4* qMSP, observations during optimisation suggest that the type of DNA template impacted the quantification and the limited Δβ -value between HC and RA in PBMC, suggests that this may also be modulated in other cells types, explaining the loss of signal. This is biologically relevant considering the role of the *HDAC4* gene in epigenetic remodelling, while it prevent this candidate to be a biomarker. Altogether, our data demonstrate that analysing the DNA region around the candidate CpG is critical to the design of the assays, while considering various blood cell-types methylation patterns will define the type of material that can be used.

SUP Table S3 : qMSP assay primer and probe details

Gene	Position	F/R/Probe	sequence	Product size
TNF	Chr 6: 31,543,091-31,543,211	F 5' to 3'	TTT**CG**GAAT**CG**GAGTAGGGAG	121
		R 5' to 3'	ACCCTACACCTTCTATCT**CG**ATTTCTT	
		Probe	T**CG**TTTT**CGCG**ATGGAG	
HDAC4	Chr 2: 240,196,872-240,196,954	F 5' to 3'	TGGGT**CG**AAGTTATTTTAGGTTTTTAGT	83
		R 5' to 3'	AA**CG**ACTTACCAAAAACAACCTCAA	
		Probe	TAGATTATTAGGT**CGCG**TAGGTT	
IRF8 (V.1)	Chr 16: 85,979,046-85,979,116	F 5' to 3'	TGAAGTAGTAGTTT**CG**GTATTGGGTTT	71
		R 5' to 3'	ACCAACCCA**CG**CCAAAAA	
		Probe	TAGTGGAGAT**CG**GGAATGA	
IRF8 (V.2)	Chr 16: 85,979,046-85,979,126	F 5' to 3'	TGAAGTAGTAGTTT**CG**GTATTGGGTTT	81
		R 5' to 3'	CTA**CG**TCCTTACCAACCCA**CG**	
		Probe	TAGTGGAGAT**CG**GGAATGA	
GAPDH	Chr 12: 6,645,449-6,645,570	F 5' to 3'	TTGGGTAGTTTTGGAGTTTTTAGTTG	122
		R 5' to 3'	AATACAACATCTCCTTACCCCCAA	
		Probe	AGTTAGGTTAGTTTGGTAGGGAA	

SUP Table S4 : qMSP final condition

Final Taq-man qMSP reaction composition and cycling conditions.

qPCR TagMan assay

Stock conc.	Reagent	Final concentration		
		GAPDH	HDAC4	TNF
2X	universal mastermixII no UNG	1X	1X	1X
vary	F primer	300 nM	900 nM	900 nM
vary	R primer	900 nM	900 nM	900 nM
2.5 uM	Taqman probe	250 nM	250 nM	250 nM
	DNA template	20 ng/uL	20 ng/uL	20 ng/uL
	Total Volumn 20 ul			

PCR cycling condition

Step	Temperature	Time	
Initial activation	95 C	10 min	
Denaturation	95 C	15 s	50 cycles
Annealing/Extension	60 C	60 s	

SUP data : qMSP calibration

The relative level of methylation in the target gene was measured using Ct value of each reactions (target and internal control). It is presented as a percentage of methylation (%). A calibrator is included one ach PCR plate to ensure reproducibility between all experiments due to the large number of samples analyzed over time. The formula to calculate this is as followed.

Percentage of methylation (%) = Relative level of methylation x 100

$$= 2^{-\Delta\Delta Ct} \times 100$$

where

$$\Delta Ct\ sample = Ct_{sample}\ target\ gene - Ct_{sample}\ internal\ control$$

$$\Delta Ct\ calibrator = Ct_{calibrator}\ target\ gene - Ct_{calibrator}\ internal\ control$$

$$\Delta\Delta Ct = \Delta Ct\ sample - \Delta Ct\ calibrator$$

Sample DNA template from an individual patient after bisulfite converted.

Calibrator Control DNA template 100% methylated and bisulfite converted

Target gene is the gene of interest for the assay (for example the TNF gene) at the CpG chose for the assay development with expected change in methylation status

Internal control is the GAPDH gene used for normalization from a region independent of any methylation change

SUP Figure S2 : % of methylation for the *HDAC4* qMSP in CD4+T-cells and PBMC.

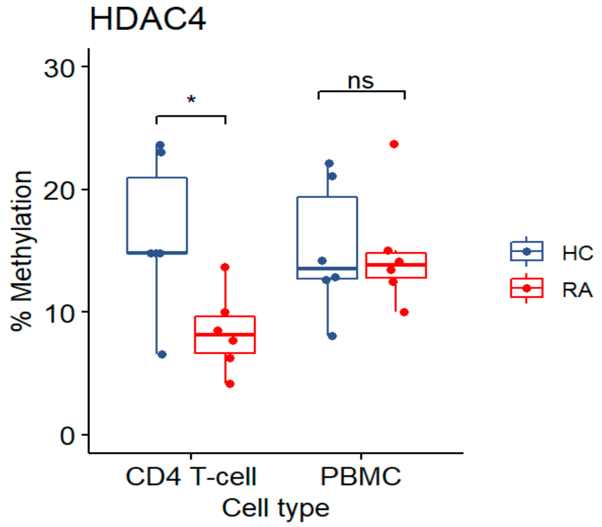

Boxplot of the % of methylation of the *HDAC4* qMSP in CD4+T-cells or PBMC in early RA (red, n=6) and healthy control (blue, n=6). * p<0.05. NS not significant.

SUP Figure S3 : HDAC4-qMSP data

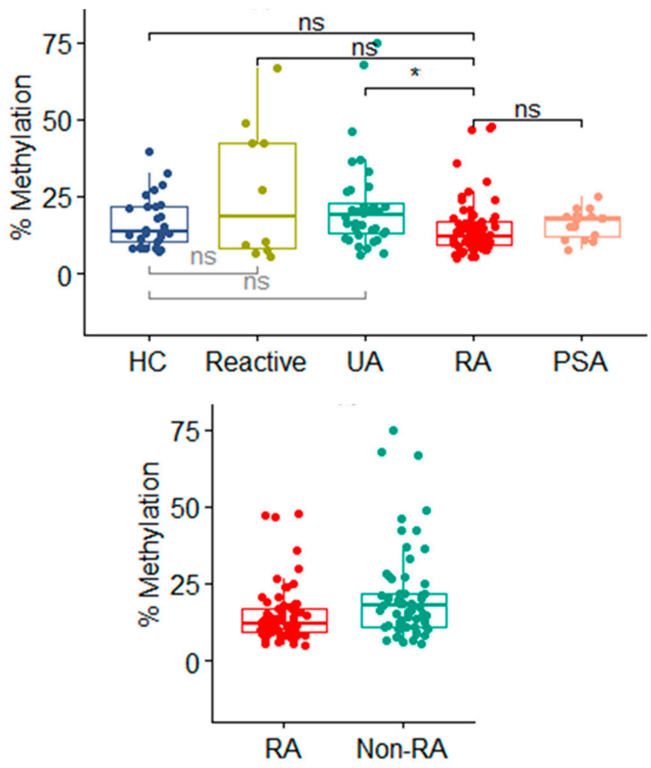

Boxplot of the % of methylation observed using the *HDAC4* qMSP in PBMC from IACON patients (n=127). *p<0.05, NS not significant. Comparing RA to non-RA, % methylation were lower in RA (p=0.0027). ______________________

SUP Table S5 : Patient characteristics in the combined dataset IACON + RADAR (top section) and in ACPA-negative patients (bottom section).

**All patients (n=284)**	RA (n=190)	Non-RA (n=94)	MWT or chi2 p-value	OR p-value	AUC (95%CI)
TNF methylation levels*	3.26 [2.48;4.37]	6.16 [4.77;7.92]	1.96×10^{-19}	0.54 [0.46; 0.64] $p = 5.57 \times 10^{-13}$	0.171 (0.115-0.227)
TNF high risk n (%)	150 (78.9%)	18 (19.1%)	$<2.2 \times 10^{-22}$	15.8 [8.51;29.5] $p = 1.0 \times 10^{-16}$	0.799 (0.750 - 0.848)
Age (years)*	58.5 [49.0;70.0]	45.0 [34.0;52.0]	8.93×10^{-12}	1.06 [1.04;1.08] $p = 3.88 \times 10^{-10}$	0.749 (0. 689,0.808)
Female fender n (%)	137 (72.1%)	65 (69.1%)	0.7053	1.15 [0.67;1.98] p = 0.6052	0.515 (0.458,0.572)
Symptoms duration (months)*	5.73 [3.21;11.9]	6.00 [3.00;11.9]	0.8103	0.99 [0.97;1.01] p = 0.2780	0.507 (0.434,0.579)
Smokers n (%)	115 (62.5%)	42 (47.7%)	0.0295	2.17 [1.31;3.59] $p = 2.55 \times 10^{-3}$	0.595 (0.534,0. 656)
RF positive n (%)	99 (52.7%)	10 (10.9%)	3.95×10^{-11}	9.53 [4.66;19.5] $p = 4.20 \times 10^{-13}$	0.713 (0.665 - 0.76)
ACPA positive n (%)	108 (58.4%)	8 (8.60%)	5.6×10^{-15}	14.5 [6.64;31.5] $p = 1.0 \times 10^{-16}$	0.744 (0.699 - 0.789)
Tender joint count*	9.50 [3.25;15.8]	3.00 [1.00;7.00]	3.55×10^{-7}	1.11 [1.06;1.15] $p = 2.06 \times 10^{-6}$	0.703 (0. 637 - 0.768)
Swollen joint count*	4.50 [2.00;9.00]	1.00 [0.00;4.00]	4.79×10^{-8}	1.22 [1.13;1.32] $p = 1.05 \times 10^{-6}$	0.7122 (0.52 - 0.772)
CRP (mg/L)*	11.3 [0.00;24.0]	2.50 [0.00;11.7]	0.0002	1.03 [1.01;1.05] $p = 7.82 \times 10^{-4}$	0.647 (0.583 - 0.712)
DAS28*	4.57 [3.51;5.49]	3.30 [2.37;4.10]	5.62×10^{-9}	2.01 [1.62;2.51] $p = 4.20 \times 10^{-10}$	0.751 (0. 692 - 0.809)
**ACPA negative patients (n=167)**	RA (n=81)	Non-RA (n=86)	MWT or chi2 p-value	OR p-value	AUC (95%CI)
TNF methylation levels*	3.02 [2.27;3.85]	6.10 [4.72;7.92]	9.8×10^{-16}	0.45 [0.34;0.58] $p = 1.15 \times 10^{-9}$	0.140 (0.079 - 0.201)

TNF high risk n (%)	70 (86.4%)	18 (20.9%)	1×10^{-16}	24.0 [10.6;54.6] $p = 2.2\times10^{-22}$	0.827 (0.770 - 0.885)
Age (years)*	64.0 [52.0;73.0]	45.0 [34.0;52.0]	7.68×10^{-13}	1.10 [1.06;1.13] $p = 6.14 \times 10^{-10}$	0.821 (0.758 - 0.884)
Female fender n (%)	64 (79.0%)	60 (69.8%)	0.2346	1.63 [0.81;3.30] p = 0.1782	0.546 (0.480 - 0.124)
Symptoms duration (months)*	5.05 [2.75;9.63]	6.00 [3.00;11.4]	0.3234	0.99 [0.96;1.01] p = 0.3355	0.544 (0.456 - 0. 632)
Smokers n (%)	48 (59.3%)	38 (44.2%)	0.0729	1.84 [0.99;3.40] p = 0.0539	0.575 (0.5 - 0. 651)
RF positive n (%)	28 (34.6%)	6 (6.98%)	2.30×10^{-5}	7.04 [2.73;18.2] $p = 7.95 \times 10^{-6}$	0. 638 (0.579 - 0. 696)
Tender joint count*	11.0 [4.00;16.0]	3.00 [2.00;6.75]	5.01×10^{-7}	1.12 [1.07;1.17] $p = 4.25 \times 10^{-6}$	0.725 (0. 645 - 0.804)
Swollen joint count*	5.50 [2.00;11.0]	1.00 [0.00;4.00]	2.78×10^{-6}	1.20 [1.10;1.30] $p = 1.84 \times 10^{-5}$	0.708 (0. 627 - 0.789)
CRP (mg/L)*	9.80 [0.00;21.0]	2.50 [0.00;10.7]	0.0017	1.03 [1.01;1.05] p = 0.0047	0.636 (0.554 - 0.717)
DAS28*	4.91 [3.48;5.64]	3.12 [2.46;3.97]	8.43×10^{-9}	2.06 [1.57;2.69] $p = 1.46 \times 10^{-7}$	0.758 (0.83 - 0.833)

Data are described using n (% of patients), * median (IQR). RF rheumatoid factor, ACPA anti-citrullinated peptide antibodies, TJC tender joints count, SJC swollen joints count, CRP C-reactive protein, DAS28 disease activity score 28 joints.

SUP Table S6 : Statistical differences for demographic and clinical parameters between cohorts (IACON and RADAR) for both RA and Non-RA groups

parameter	RA (IACON n=64, RADAR n= 126)	Non-RA (IACON n=63, RADAR n= 31)
TNF methylation levels*	p = 0.3621	p = 0.0845
Age * (years)	p = 0.4409	p = 0.0154
Female fender n (%)	p = 0.1363	p = 0.6565
Symptoms Duration* (months)	p = 0.0996	p = 0.7891
Smokers n (%)	p = 0.5159	p = 0.4463
RF positive (%)	p = 0.4972	p = 0.9263
ACPA positive (%)	p = 0.0782	p = 0.0209
Tender joint count*	p = 0.2971	p = 0.0729
Swollen joint count*	p = 0.5992	p = 0.0031
CRP * (mg/L)	p = 0.6644	p = 0.7453
DAS28*	p = 0.0651	p = 0.0270

Supp **Table S7.** : Binary logistic regression (n=284) Backward method.

OR (95% CI) p value	Unadjusted	Reference Model	TNF qMSP model (levels)
TNF methylation levels	0.54 (0.46, 0.64) <0.0001		0.50 (0.39, 0.65) <0.0001
Age	1.06 (1.04, 1.08) <0.0001	1.064 (1.03, 1.09) <0.0001	1.06 (1.025, 1.09) 0.001
Female gender	1.15 (0.67, 1.98) 0.6052	not selected	2.89 (0.999, 8.39) 0.052
RF positive	9.53 (4.66, 19.5) <0.0001	4.40 (1.63, 12.51) 0.003	2.85 (1.07, 8.78) 0.066
ACPA positive	14.5 (6.64, 31.5) <0.0001	22.72 (7.40, 71.45) <0.0001	45.5 (11.25, 200) <0.0001
DAS28	2.01 (1.62, 2.51) <0.0001	1.80 (1.33, 2.44) <0.0001	1.54 (1.09, 2.17) 0.001
Accuracy (% of correctly predicted cases)		85.51	90.10
AUC (95% CI)		0.911 (0.882, 0.950)	0.946 (0.920, 0.973)

RF rheumatoid factor, ACPA anti-citrullinated peptide antibodies, DAS28 disease activity score 28 joints. AUC area under the ROC curve, CI confidence interval.

References

49. Rodriguez A, Rodriguez M, Cordoba JJ, Andrade MJ. Design of primers and probes for quantitative real-time PCR methods. Methods Mol Biol. 2015;1275:31-56.
50. Davidović R, Božović A, Mandušić V, Krajnović M. Methylation-specific PCR: four steps in primer design. Open Life Sciences. 2014;9(12):1127-39.
51. AppliedBiosystems. Real-time PCR handbook2014. Available from: https://www.thermofisher.com/content/dam/LifeTech/global/Forms/PDF/real-time-pcr-handbook.pdf.
52. AppliedBiosystem. Designing TaqMan® MGB Probe and Primer Sets for Allelic Discrimination Assays Using Primer Express®Software 2015. Available from: https://www.thermofisher.com/document-connect/document-connect.html?url=https://assets.thermofisher.com/TFS-Assets%2FLSG%2Fmanuals%2Fcms_041902.pdf.
53. Ouyang X, Zhang R, Yang J, Li Q, Qin L, Zhu C, et al. Transcription factor IRF8 directs a silencing programme for TH17 cell differentiation. Nat Commun. 2011;2:314.
54. Yu H, Lu C, Tan MT, Moudgil KD. The gene expression profile of preclinical autoimmune arthritis and its modulation by a tolerogenic disease-protective antigenic challenge. Arthritis Res Ther. 2011;13(5):R143.
55. Ouboussad L, Hunt L, Hensor EMA, Nam JL, Barnes NA, Emery P, et al. Profiling microRNAs in individuals at risk of progression to rheumatoid arthritis. Arthritis Res Ther. 2017;19(1):288.
56. Dong L, Wang X, Tan J, Li H, Qian W, Chen J, et al. Decreased expression of microRNA-21 correlates with the imbalance of Th17 and Treg cells in patients with rheumatoid arthritis. J Cell Mol Med. 2014;18(11):2213-24.
57. Huang Z, Xing S, Liu M, Deng W, Wang Y, Huang Z, et al. MiR-26a-5p enhances cells proliferation, invasion, and apoptosis resistance of fibroblast-like synoviocytes in rheumatoid arthritis by regulating PTEN/PI3K/AKT pathway. Biosci Rep. 2019;39(7).
58. Shengwei J, Huaijun C, Yongsheng L, Hao Z, Weiwei S, Jianmin W, et al. Maresin 1 improves the Treg/Th17 imbalance in rheumatoid arthritis through miR-21. Annals of the Rheumatic Diseases. 2018;77(11):1644.
59. Deng Y, Zhou Y, Liang Q, Ge C, Yang J, Shan B, et al. Inflammation-Instructed Hierarchical Delivery of IL-4/miR-21 Orchestrates Osteoimmune Microenvironment toward the Treatment of Rheumatoid Arthritis. Advanced Functional Materials. 2021;31(33):2101033.
60. Churov AV, Oleinik EK, Knip M. MicroRNAs in rheumatoid arthritis: Altered expression and diagnostic potential. Autoimmunity Reviews. 2015;14(11):1029-37.
